# Dentoalveolar and skeletal changes after customized elastodontic appliance therapy in mixed dentition children with maxillary transverse deficiency: a retrospective CBCT study

**DOI:** 10.1186/s12903-026-09622-7

**Published:** 2026-08-15

**Authors:** Jianhua Li, Xinran Fan, Qingyu Wang

**Affiliations:** 1 Hangzhou Stomatology Hospital, Hangzhou, China; 2 Suzhou Industrial Park Chunlu Dental Clinic, Suzhou, China

**Keywords:** Customized elastodontic appliance, Expansion, Cone-beam computed tomography, Mixed dentition, Dentoalveolar changes, Transverse deficiency

## Abstract

**Background:**

This exploratory study aimed to evaluate the expansion effects of a customized elastodontic appliance (CEA) on maxillary and mandibular arches in mixed dentition children using cone-beam computed tomography (CBCT), and to clarify whether these effects are dental, dentoalveolar, or skeletal.

**Methods:**

This retrospective study included 31 mixed dentition children (mean age 8.3 ± 1.2 years) who underwent CEA treatment for 8.2 ± 1.3 months. Pre- and post-treatment CBCT images were used to measure width changes at three levels: cusp, alveolar bone, and apical basal bone. Paired t-tests were used for comparisons.

**Results:**

Significant increases were observed at cusp and alveolar levels. At the cusp level, maxillary inter-canine width increased by 2.97 mm ± 1.31 mm (95% CI: 2.49–3.45, *P* < 0.001), and mandibular inter-canine width by 2.39 mm ± 1.32 mm (95% CI: 1.91–2.87, *P* < 0.001). Maxillary second primary molar buccal cusp width increased by 1.67 mm **±** 0.85 mm (95% CI: 1.36–1.98, *P* < 0.001), and mandibular counterpart by 1.26 mm **±** 1.11 mm (95% CI: 0.85–1.67, *P* < 0.001). At the alveolar bone level, maxillary alveolar crest width increased by 1.39 mm ± 1.09 mm (95% CI: 0.99–1.79, *P* < 0.001), whereas mandibular alveolar crest width showed a minimal increase of 0.38 mm ± 0.75 mm (95% CI: 0.10–0.66, *P* = 0.008). No significant changes were observed in apical basal bone width in either arch.

**Conclusion:**

The findings suggest that CEA may expand maxillary and mandibular arches in mixed dentition children with maxillary transverse deficiency. The maxillary expansion effect primarily involves dental movement and adaptive dentoalveolar change, without significant deep basal bone changes. In the mandible, significant dental expansion was observed at the cusp level, while the alveolar bone changes were minimal and should be interpreted cautiously. These findings provide preliminary CBCT-based evidence for the transverse expansion effects of CEA in mixed dentition children.

**Clinical trial registration:**

Not applicable. This is a retrospective, anonymous observational study without prospective intervention or a clinical trial protocol.

## Background

Adequate dental arch width development is the cornerstone of early malocclusion correction in children. In proposing the six keys to normal occlusion, Andrews identified harmonious arch form as a core element [1]. Maxillary transverse deficiency not only directly leads to local problems such as dental crowding and posterior crossbite [2] but also exerts profound effects on the overall development of the craniofacial skeleton. Sagittally, a constricted maxillary arch can restrict the mandible to a retruded position, interfering with its normal forward growth, thereby inducing or exacerbating functional mandibular retrognathia—a significant contributing factor to Angle Class II malocclusion [3]. Vertically, arch constriction often accompanies a high-arched palate, potentially encroaching on the nasal floor space and compromising upper airway patency. Therefore, effective arch width expansion not only resolves dental crowding but, more importantly, removes restrictions on mandibular growth, coordinates maxillomandibular relationships, and creates conditions for establishing normal respiratory function. Based on this, the “width-first” correction strategy advocates first establishing a coordinated platform in three dimensions for the normal development of the craniofacial skeleton and dentition, rendering subsequent orthodontic treatment more efficient, stable, and aligned with physiological growth patterns [4].

The advent of cone-beam computed tomography (CBCT) has ushered in a new era of three-dimensional precision in arch width research, moving beyond traditional two-dimensional plaster model measurements. Researchers can now non-invasively and accurately distinguish dental expansion (buccal tooth inclination) from skeletal expansion (basal bone and midpalatal suture changes), while simultaneously evaluating adaptive changes in adjacent structures such as the nasal cavity and alveolar bone. Since Baumgaertel et al. [5] systematically validated the reliability and accuracy of CBCT dental measurements, this technology has become the gold standard tool for studying the width effects of various orthodontic appliances, with subsequent systematic reviews confirming its reliability in transverse diagnosis [6]. Multiple studies have utilized CBCT to precisely quantify the skeletal and dental width changes produced by different appliances [7, 8], providing unprecedented depth for clinical efficacy evaluation.

Among the numerous early orthodontic interventions, elastodontic appliances (EAs, silicone-based functional appliances) have gained widespread clinical attention and application due to their unique mechanism of action [9–11]. Unlike traditional appliances that simply apply mechanical force, EAs exert effects through a dual pathway: on one hand, the appliance encapsulates the clinical crowns and extends to the lingual alveolar bone, where the sustained force generated by silicone deformation during wear directly drives arch expansion at both the crown and alveolar bone levels; on the other hand, the buccal shields physically isolate abnormal pressure from the buccinator and masseter muscles, while the tongue ramp guides the tongue to its correct position, achieving a rebalancing of orofacial musculature, thereby promoting physiological arch expansion [12–15].

EAs can be classified into preformed and customized elastodontic appliance (CEA) types. Preformed EAs offer economic advantages, whereas CEAs, customized based on patients’ intraoral scan data, provide superior adaptation and theoretically produce more precise and stable clinical outcomes [16–19]. However, current research on CEAs has primarily focused on sagittal and vertical correction effects [16, 18–19], with systematic quantification of their transverse expansion effects remaining limited. More critically, previous studies have failed to utilize CBCT, the gold standard technique, to distinguish whether the expansion effect of CEAs originates from buccal tooth movement, adaptive alveolar bone remodeling, or structural widening of the basal bone. In other words, the three-dimensional mechanism of CEA-induced arch expansion in the canine and molar regions of the maxilla and mandible still lacks rigorous imaging evidence.

Given the lack of CBCT evidence on the mechanism of CEA-induced expansion, this exploratory study aimed to quantitatively evaluate the effect of CEA on arch width in mixed dentition children through a retrospective CBCT study, clarify the nature (dental/ dentoalveolar /skeletal), magnitude, and three-dimensional characteristics of its therapeutic effects, and provide precise imaging evidence to inform clinical decision-making.

## Methods

### Study design and participants

This retrospective study aimed to evaluate the effect of CEA on arch width in mixed dentition children. All consecutive children who completed CEA treatment at Hangzhou Stomatology Hospital and Suzhou Chunlu Dental Clinic between February 2023 and June 2025 and met the eligibility criteria were included.

Inclusion criteria: (1) early mixed dentition with fully erupted and stable maxillary and mandibular second primary molars; (2) clinical signs of maxillary transverse deficiency (e.g., unilateral or bilateral posterior crossbite, narrow maxillary arch with a high-arched palate, or dental crowding requiring arch expansion to gain space); (3) no history of maxillofacial trauma or systemic/genetic diseases affecting jaw growth; (4) symmetrical face with no obvious skeletal deformity; (5) healthy periodontium; (6) availability of CBCT images of satisfactory quality for measurement both before (T0) and after (T1) treatment; (7) good treatment compliance, confirmed by follow-up interviews and appliance wear assessment.

Exclusion criteria: (1) history of orthodontic treatment; (2) premature loss of primary teeth or congenitally missing permanent teeth; (3) presence of dental developmental abnormalities.

According to the above criteria, 31 patients were finally included, comprising 17 males and 14 females, with a mean age of 8.3 ± 1.2 years. All patients were in early mixed dentition with fully erupted and stable maxillary and mandibular second primary molars, which were therefore used as the reference landmarks for all posterior width measurements. The mean treatment duration was 8.2 ± 1.3 months.

A formal sample size calculation was not performed for this exploratory retrospective study. The sample size (*n* = 31) was determined by the availability of eligible patients during the study period with complete pre- and post-treatment CBCT records. All consecutive patients who met the inclusion criteria were included. A post-hoc power analysis was subsequently performed to assess whether the obtained sample size was adequate to detect the observed treatment effects. The details and results are presented in the Results section.

All methods were performed in accordance with the relevant guidelines and regulations of Declaration of Helsinki. This retrospective study was approved by the Medical Ethics Committee of Hangzhou Stomatology Hospital (Approval No. 2025LL04, approved on August 18, 2025), covering patient data from both centers (Suzhou Chunlu Dental Clinic lacked an independent ethics committee and relied on Hangzhou center’s approval). A written informed consent to participate was obtained from all subjects and/or their legal guardian(s). All data were retrospectively and anonymously analyzed.

### Appliance design and treatment protocol

**Fig. 1:**
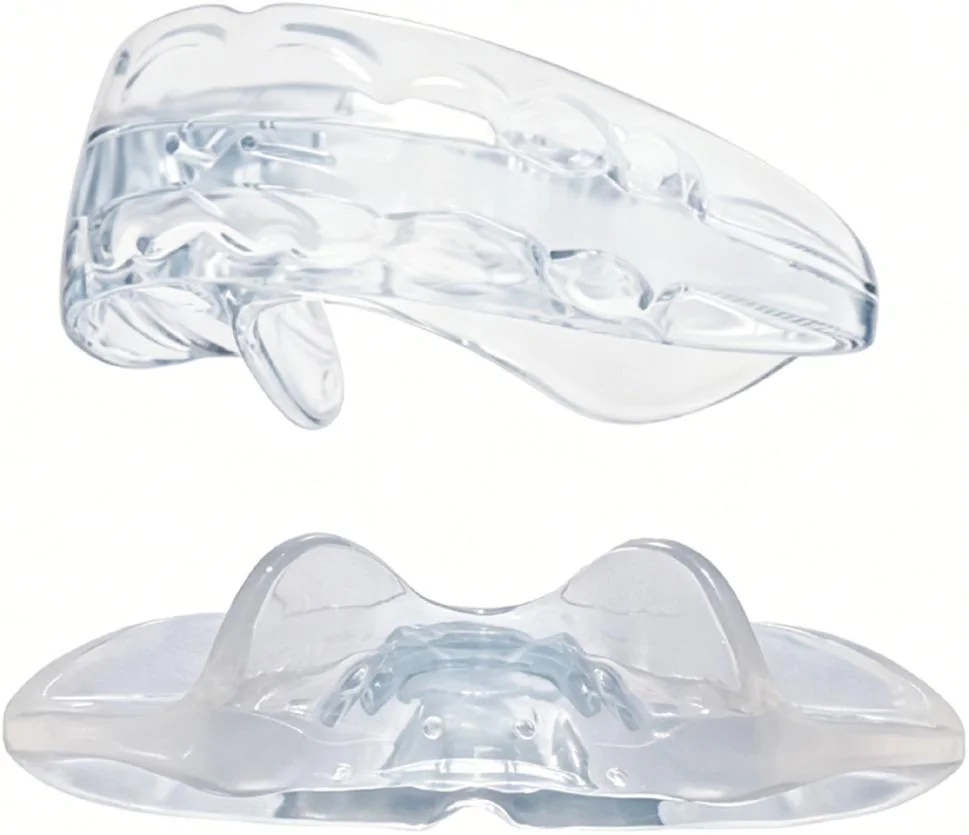
Customized elastodontic appliance (ETA brand, BJ Appliance Health Science & Technology Co., Ltd., China)

The CEA appliances (BJ Appliance Health Science & Technology Co., Ltd., China) were designed and manufactured according to a standardized digital workflow based on comprehensive analysis of intraoral scan data, radiographs, and intraoral and extraoral photographs (Fig. 1).

The treatment protocol was as follows: patients were instructed to wear the appliance cumulatively for more than 2 h during the daytime and throughout the night, with follow-up visits every 4–6 weeks. The treatment endpoint was defined as: resolution of posterior crossbite, improvement of arch constriction signs, or achievement of the prespecified arch form goal. Active treatment was terminated upon reaching the treatment endpoint. The mean treatment duration for this cohort was 8.2 ± 1.3 months.

Treatment at the two centers was performed by the two authors of this study, both senior orthodontic specialists with > 15 years of clinical experience, maintaining close academic collaboration and a high degree of consistency in CEA treatment philosophy and clinical protocols. Retrospective verification confirmed that the two centers were consistent in wearing protocol, follow-up intervals, and treatment endpoint criteria.

### CBCT image acquisition

Pre-treatment (T0) and post-treatment (T1) CBCT images were acquired as part of routine clinical assessment and treatment evaluation. Both scans were performed using KaVo CBCT devices (one OP 3D Pro, the other X-trend, KaVo Dental GmbH, Germany), with uniformly set low-dose paediatric parameters: 90 kV, 5 mA, field of view 12 × 15 cm, and metal artifact reduction (MAR) activated. All scans were performed with the patient in natural head posture and the teeth in maximum intercuspation, in accordance with the ALARA principle.

The two devices demonstrated consistency in key performance parameters under the same scanning settings: tube voltage range 57–90 kV, tube current 3.2–16 mA, voxel size 85–420 μm, both equipped with CMOS image sensors and MAR function. To control for inter-device variability, all CBCT data were reconstructed and measured by the same investigator using the same software (OnDemand3D CD Viewer, Cybermed Inc., Korea) on the same workstation. The positioning of measurement reference points uniformly followed the definitions in Table 1.

**Table 1:** Measured items in CBCT

Abbreviation	Measured item	Description	Cross-section
MC-CW	Maxillary canine-cusp width	The distance between the cusps of bilateral maxillary (deciduous) canines, representing the width of the anterior segment of the maxillary dental arch	The coronal plane of the cusp of the maxillary (deciduous) canine
MSPM-BCW	Maxillary second primary molar-buccal cusp width	The distance between the buccal cusps of bilateral maxillary second primary molars, representing the width of the posterior segment of the maxillary dental arch	The coronal plane of the buccal cusp of the maxillary second primary molar
MSPM-ACW	Maxillary second primary molar-alveolar crest width	The distance between the buccal alveolar crest of the maxillary bilateral second primary molar, representing the width of the posterior segment of the maxillary alveolar bone arch	The coronal plane of most convex point of the buccal alveolar crest of the bilateral second primary molar
MSPM-BBW	Maxillary second primary molar-basal bone width	Width of the buccal basal bone at the apical level of the second primary molars, representing the width of the posterior segment of the maxillary basal bone arch	The coronal plane of most concave point of the buccal cortical bone at the root apical level of the second primary molars
LC-CW	Lower canine-cusp width	The distance between the cusps of bilateral mandibular (deciduous) canines, representing the width of the anterior segment of the mandibular dental arch	The coronal plane of the cusp of the mandibular (deciduous) canine
LSPM-BCW	Lower second primary molar- buccal cusp width	The distance between the cusps of bilateral mandibular second primary molars, representing the width of the posterior segment of the mandibular dental arch	The coronal plane of the buccal cusp of the mandibular second primary molar
LSPM-ACW	Lower second primary molar-alveolar crest width	The distance between the buccal alveolar crest of the mandibular bilateral second primary molars, representing the width of the posterior segment of the mandibular alveolar bone arch	The coronal plane of most convex point of the buccal alveolar crest of the bilateral second primary molar
LSPM-BBW	Lower second primary molar-basal bone width	Width of the buccal basal bone at the apical level of the second primary molars, representing the width of the posterior segment of the mandibular basal bone arch	The coronal plane of most concave point of the buccal cortical bone at the root apical level of the second primary molars

### Blinding procedure

To ensure objectivity during the measurement process, all CBCT images were measured under blinded conditions. An independent assistant not involved in the study randomly renumbered all scans (including both pre-treatment and post-treatment images) using a random number generator, so that the investigator was completely unaware of the time point (T0 vs. T1), patient identity, and treatment center throughout the entire measurement process. The mapping between the new codes and original identifiers was kept by the assistant and was not disclosed to the investigator until all measurements were completed. After the measurements, the assistant decoded the data and reassembled them into pre- and post-treatment pairs for statistical analysis.

### Measurement parameters and localization method

**Fig. 2:**
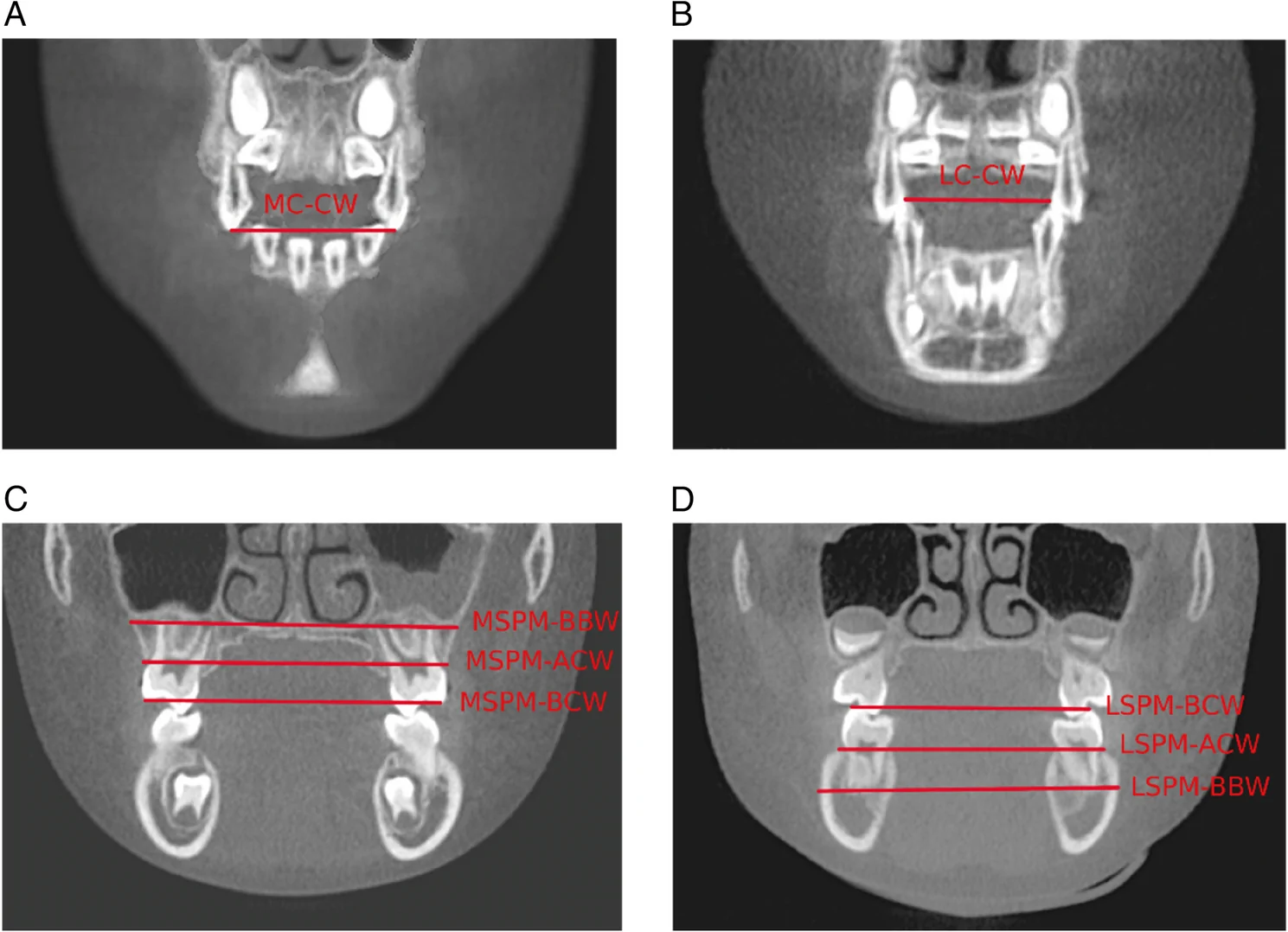
Measured items. **A **MC-CW (Maxillary canine-cusp width); **B **LC-CW (Lower canine-cusp width); **C **MSPM-BCW (Maxillary second primary molar-buccal cusp width), MSPM-ACW (Maxillary second primary molar-alveolar crest width), MSPM-BBW (Maxillary second primary molar-basal bone width); **D **LSPM-BCW (Lower second primary molar-buccal cusp width), LSPM-ACW (Lower second primary molar-alveolar crest width), LSPM-BBW (Lower second primary molar-basal bone width)

Given the variable eruption status of first permanent molars in early mixed dentition patients, this study uniformly used the maxillary and mandibular second primary molars as the reference landmarks for all posterior width measurements. The measurement parameters comprised 8 parameters across three levels (Table 1; Fig. 2):

- *Cusp level*: Maxillary and mandibular inter-canine cusp width (MC-CW, LC-CW), maxillary and mandibular second primary molar buccal cusp width (MSPM-BCW, LSPM-BCW)
- *Alveolar bone level*: Maxillary and mandibular buccal alveolar crest width at the second primary molar region (MSPM-ACW, LSPM-ACW)
- *Basal bone level*: Maxillary and mandibular buccal basal bone width at the root apex level of the second primary molar (MSPM-BBW, LSPM-BBW)

Detailed measurement methods for each parameter are presented in Table 1; Fig. 2. In brief, cusp width was measured as the linear distance between the corresponding cusp tips bilaterally; alveolar crest width was measured as the linear distance between the most convex points of the buccal alveolar crest of the bilateral second primary molars; apical basal bone width was measured as the linear distance between the most concave points of the buccal cortical bone at the root apex level of the bilateral second primary molars. All second primary molars remained clinically stable throughout the treatment period without mobility or exfoliation. In cases where the root apex was no longer clearly discernible on the post-treatment images due to physiological root resorption, the measurement was performed at the corresponding anatomical level determined from the pre-treatment scan to ensure comparability between time points.

### Intra-examiner reliability

To assess intra-examiner reliability, 15 patients (48.4% of the total sample) were randomly selected. The investigator re-measured all parameters for these patients in a second session, conducted two weeks after the initial measurements under identical blinded conditions. The intraclass correlation coefficient (ICC) was calculated to evaluate the consistency between the two sets of measurements.

### Statistical analysis

Statistical analyses were performed using SPSS version 25.0 (IBM Corp., Armonk, NY, USA). All continuous data were expressed as mean ± standard deviation (x̄ ± s). Intra-examiner reliability was assessed using the intraclass correlation coefficient (ICC) with a two-way random-effects model for absolute agreement.

#### Baseline comparability between centers

Independent samples t-test (for continuous variables) or chi-square test (for categorical variables) was used to compare baseline characteristics (age, sex, treatment duration, and all initial arch width measurements) between the two centers, to verify whether data from the two centers could be combined for analysis.

#### Normality test

The Shapiro-Wilk test was used to test the normality of the pre-post treatment differences (Δ = T1 – T0) for each measurement parameter to determine whether parametric or nonparametric tests should be used.

#### Overall pre-post treatment comparison (combined data)

After combining data from both centers (*n* = 31), paired t-tests (if data were normally distributed) or Wilcoxon signed-rank tests (if data were not normally distributed) were used to compare differences in each measurement parameter before and after treatment, to evaluate the overall efficacy of CEA.

#### Center-specific pre-post treatment comparison (sensitivity analysis)

For the two centers separately, paired t-tests or Wilcoxon signed-rank tests were used to compare differences in each measurement parameter before and after treatment, to verify the robustness of the treatment effect across centers.

#### Comparison of treatment effects between centers (reproducibility analysis)

Independent samples t-tests were used to compare the changes in each parameter between the two centers, to assess the reproducibility of CEA efficacy in different clinical settings.

A P-value < 0.05 was considered statistically significant. All statistical tests were two-tailed.

To assess the robustness of the findings, sensitivity analyses using the Wilcoxon signed-rank test were performed for all outcome measures.

No adjustment for multiple comparisons was applied because the outcome measures were correlated and the study was exploratory. Results should be interpreted based on effect sizes and their 95% confidence intervals.

## Results

### Study sample and baseline characteristics

A total of 31 mixed dentition children were included in this study, comprising 17 males (54.8%) and 14 females (45.2%), with a mean age of 8.3 ± 1.2 years and a mean treatment duration of 8.2 ± 1.3 months. Hangzhou Stomatology Hospital contributed 17 cases (54.8%), and Chunlu Dental Clinic contributed 14 cases (45.2%).

#### Intra-examiner reliability

Intra-examiner reliability analysis showed that the intraclass correlation coefficients (ICC) for all measurement parameters exceeded 0.95 (95% CI: 0.92–0.98), indicating excellent intra-examiner reliability.

#### Baseline comparability between centers

Independent samples t-test and chi-square test showed no statistically significant differences between the two centers in age, sex, treatment duration, or any baseline arch width measurements (all P > 0.05, Table 2), indicating good comparability between the two centers and justifying the combination of data for subsequent analyses.

**Table 2:** Comparison of baseline characteristics between the two centers (mean ± SD)

Variable	Hangzhou center (*n* = 17)	Suzhou center (*n* = 14)	*P* value
Age (years)	7.94 ± 1.09	8.43 ± 1.22	0.246
Treatment duration (months)	7.88 ± 1.45	8.43 ± 1.16	0.257
Sex (male/female)	10/7	7/7	0.56
Maxillary arch (T0, mm)			
MC-CW	31.02 ± 2.41	32.35 ± 1.88	0.102
MSPM-BCW	46.83 ± 1.09	47.28 ± 1.15	0.24
MSPM-ACW	50.33 ± 1.04	51.04 ± 1.26	0.096
MSPM-BBW	57.09 ± 1.16	57.86 ± 2.11	0.204
Mandibular arch (T0, mm)			
LC-CW	22.61 ± 2.27	23.67 ± 2.01	0.184
LSPM-BCW	41.05 ± 1.23	41.96 ± 1.75	0.101
LSPM-ACW	46.97 ± 1.12	47.67 ± 1.73	0.173
LSPM-BBW	54.59 ± 2.49	55.25 ± 2.07	0.429

#### Normality test

Shapiro-Wilk tests on pre-post treatment differences (Δ = T1 – T0) for all measurement parameters yielded P-values > 0.05, indicating that the data were normally distributed. Therefore, parametric tests (paired t-tests) were used for all subsequent pre-post treatment comparisons.

### Arch width changes: overall efficacy analysis

After combining data from both centers (*n* = 31), the statistical results for arch width changes before and after treatment are presented in Table 3. Paired t-test showed:

- *Cusp level*: Maxillary inter-canine width increased by 2.97 mm (95% CI: 2.49–3.45, P < 0.001), and mandibular inter-canine width by 2.39 mm (95% CI: 1.91–2.87, P < 0.001). Maxillary second primary molar buccal cusp width increased by 1.67 mm (95% CI: 1.36–1.98, P < 0.001), and mandibular counterpart by 1.26 mm (95% CI: 0.85–1.67, P < 0.001).
- *Alveolar bone level*: Maxillary alveolar crest width in the second primary molar region increased by 1.39 ± 1.09 mm (95% CI: 0.99 to 1.79, P < 0.001), which was largely comparable to the corresponding cusp-level expansion (1.67 ± 0.85 mm). The mandibular alveolar crest width also showed a statistically significant increase of 0.38 ± 0.75 mm (95% CI: 0.10 to 0.66, P = 0.008), but its small magnitude warrants cautious interpretation.
- *Basal bone level*: No significant changes were observed in apical basal bone width in either the maxilla (0.21 mm, 95% CI: − 0.15 to 0.57, P = 0.298) or the mandible (–0.08 mm, 95% CI: − 0.40 to 0.24, P = 0.613).
- *Sensitivity analysis*: The Wilcoxon signed-rank test yielded significance conclusions for all parameters that were identical to those obtained with the paired t-tests, confirming the robustness of the findings.

These results indicate that CEA treatment significantly increased maxillary and mandibular arch widths, suggesting that the expansion effect primarily involves cusp-level dental expansion and adaptive dentoalveolar change, without significant widening of the deep basal bone.

**Table 3:** Changes in arch width measurements in the combined sample (mm, mean ± SD)

Measurement item	Combined (*n* = 31)				
	T0	T1	ΔT ± SD	95% CI	P*
Maxillary arch					
MC-CW	31.63 ± 2.29	34.59 ± 2.21	2.97 ± 1.31	(2.49, 3.45)	< 0.001
MSPM-BCW	47.04 ± 1.15	48.72 ± 1.27	1.67 ± 0.85	(1.36, 1.98)	< 0.001
MSPM-ACW	50.65 ± 1.21	52.04 ± 1.40	1.39 ± 1.09	(0.99, 1.79)	< 0.001
MSPM-BBW	57.44 ± 1.71	57.64 ± 1.69	0.21 ± 0.99	(-0.15, 0.57)	0.298
Mandibular arch					
LC-CW	23.09 ± 2.24	25.48 ± 2.29	2.39 ± 1.32	(1.91, 2.87)	< 0.001
LSPM-BCW	41.46 ± 1.58	42.72 ± 1.74	1.26 ± 1.11	(0.85, 1.67)	< 0.001
LSPM-ACW	47.29 ± 1.52	47.67 ± 1.57	0.38 ± 0.75	(0.10, 0.66)	0.008
LSPM-BBW	54.89 ± 2.41	54.81 ± 2.58	-0.08 ± 0.87	(-0.40, 0.24)	0.613

ΔT = mean change from T0 to T1 (T1 minus T0). 95% CI = 95% confidence interval for ΔT. P* values from paired t-tests

### Arch width changes: center-specific efficacy analysis

Center-specific analyses (Table 4) showed good consistency in CEA treatment effects across the two centers:

- *Maxillary arch*: In the Hangzhou center, MC-CW increased by 2.89 ± 1.46 mm (95% CI: 2.14 to 3.64, P < 0.001), MSPM-BCW by 1.53 ± 0.82 mm (95% CI: 1.11 to 1.95, P < 0.001), and MSPM-ACW by 1.36 ± 0.86 mm (95% CI: 0.92 to 1.80, P < 0.001). The Suzhou center showed a similar trend: MC-CW increased by 3.07 ± 1.15 mm (95% CI: 2.41 to 3.73, P < 0.001), MSPM-BCW by 1.85 ± 0.86 mm (95% CI: 1.35 to 2.35, P < 0.001), and MSPM-ACW by 1.43 ± 1.36 mm (95% CI: 0.65 to 2.21, P < 0.001). Maxillary basal bone width (MSPM-BBW) showed no significant change in either center (P = 0.372 and P = 0.396).
- *Mandibular arch*: In the Hangzhou center, LC-CW increased by 2.45 ± 1.44 mm (95% CI: 1.71 to 3.19, P < 0.001), LSPM-BCW by 1.08 ± 1.15 mm (95% CI: 0.49 to 1.67, P < 0.001), while LSPM-ACW increased by 0.28 ± 0.72 mm (95% CI: -0.09 to 0.65, P = 0.129). In the Suzhou center, LC-CW increased by 2.30 ± 1.21 mm (95% CI: 1.60 to 3.00, P < 0.001), LSPM-BCW by 1.47 ± 1.11 mm (95% CI: 0.83 to 2.11, P < 0.001), and LSPM-ACW by 0.49 ± 0.81 mm (95% CI: 0.02 to 0.96, P = 0.041). Mandibular basal bone width (LSPM-BBW) showed no significant change in either center (P = 0.193 and P = 0.203).

**Table 4:** Center-specific analysis and inter-center comparison of arch width changes (mm, mean ± SD)

Measurement item	Hangzhou center (*n* = 17)					Suzhou center (*n* = 14)					Inter-center comparison
	T0	T1	ΔT ± SD	95% CI	P*	T0	T1	ΔT ± SD	95% CI	P*	*P*‡
Maxillary arch											
MC-CW	31.02 ± 2.41	33.91 ± 2.19	2.89 ± 1.46	(2.14, 3.64)	< 0.001	32.35 ± 1.88	35.42 ± 2.08	3.07 ± 1.15	(2.41, 3.73)	< 0.001	0.704
MSPM-BCW	46.83 ± 1.09	48.35 ± 1.06	1.53 ± 0.82	(1.11, 1.95)	< 0.001	47.28 ± 1.15	49.13 ± 1.23	1.85 ± 0.86	(1.35, 2.35)	< 0.001	0.298
MSPM-ACW	50.33 ± 1.04	51.69 ± 1.12	1.36 ± 0.86	(0.92, 1.80)	< 0.001	51.04 ± 1.26	52.47 ± 1.53	1.43 ± 1.36	(0.65, 2.21)	< 0.001	0.865
MSPM-BBW	57.09 ± 1.16	57.33 ± 1.40	0.24 ± 1.22	(-0.39, 0.87)	0.372	57.86 ± 2.11	58.02 ± 1.83	0.16 ± 0.65	(-0.22, 0.54)	0.396	0.827
Mandibular arch											
LC-CW	22.61 ± 2.27	25.07 ± 2.58	2.45 ± 1.44	(1.71, 3.19)	< 0.001	23.67 ± 2.01	25.98 ± 1.75	2.30 ± 1.21	(1.60, 3.00)	< 0.001	0.759
LSPM-BCW	41.05 ± 1.23	42.13 ± 1.29	1.08 ± 1.15	(0.49, 1.67)	< 0.001	41.96 ± 1.75	43.43 ± 2.02	1.47 ± 1.11	(0.83, 2.11)	< 0.001	0.325
LSPM-ACW	46.97 ± 1.12	47.26 ± 1.16	0.28 ± 0.72	(-0.09, 0.65)	0.129	47.67 ± 1.73	48.16 ± 1.81	0.49 ± 0.81	(0.02, 0.96)	0.041	0.449
LSPM-BBW	54.59 ± 2.49	54.25 ± 2.82	-0.34 ± 1.03	(-0.87, 0.19)	0.193	55.25 ± 2.07	55.49 ± 2.19	0.24 ± 0.67	(-0.15, 0.63)	0.203	0.080

Data are presented as mean ± standard deviation (SD). ΔT = mean change from T0 to T1 (T1 minus T0). 95% CI = 95% confidence interval for ΔT. P* values from paired t-tests (pre-treatment vs. post-treatment within each center). P‡ values from independent t-tests comparing ΔT between the two centers

Of note, the mandibular alveolar bone parameter (LSPM-ACW) did not reach statistical significance in the Hangzhou center (*P* = 0.129) and its 95% CI crossed zero (-0.09 to 0.65), whereas it was significant in the Suzhou center (*P* = 0.041) with a 95% CI of 0.02 to 0.96 that did not include zero. In the combined analysis (*n* = 31), the 95% CI was 0.10 to 0.66, also excluding zero. This inter-center inconsistency suggests that the mandibular alveolar effect was modest and not robust, and the significance in the combined analysis should be interpreted with caution.

### Comparison of treatment effects between centers

Independent samples t-tests (Table 4, P‡ column) showed no statistically significant differences between the two centers for any parameter (all *P* > 0.05), indicating good overall consistency of treatment effects. For LSPM-BBW, the P-value for the comparison between the increase in the Suzhou center (0.24 ± 0.67 mm) and the change in the Hangzhou center (-0.34 ± 1.03 mm) was 0.08. While not reaching statistical significance, the absolute values of the basal bone changes in both groups were small (< 0.5 mm), suggesting limited clinical significance.

### Post-hoc power analysis

A post‑hoc power analysis was performed to verify whether the sample size was adequate to detect the primary treatment effects. The analysis was based on the change in maxillary second primary molar alveolar crest width (MSPM‑ACW), which was selected as the primary outcome measure because it directly reflects alveolar bone remodeling—the key mechanism distinguishing dental from dentoalveolar and skeletal expansion. It also had a relatively modest effect size (Cohen’s dz = 1.28) among the statistically significant outcome measures; if the sample size was sufficient for this parameter, it would be adequate for all other measures with larger effect sizes. Using the observed effect size, with α = 0.05 and power = 0.8, the required sample size was estimated to be 5 participants (G*Power 3.1, two‑tailed paired t‑test). The actual sample size (*n* = 31) substantially exceeded this requirement, indicating that the study was adequately powered for the primary analysis.

## Discussion

The clinical evidence on the expansion effects of CEA remains limited, and to our knowledge, no previous CBCT study has systematically distinguished the three levels of expansion—dental, dentoalveolar, and skeletal—induced by this relatively new customized appliance. This retrospective study used CBCT-based three-dimensional measurements to address this gap in early mixed dentition children. The results showed that within a mean treatment period of 8.2 months, CEA significantly increased arch widths in the canine and molar regions of both arches and induced adaptive dentoalveolar remodeling, without significant changes in deep basal bone width.

This study observed a significant dental expansion effect. Combined data analysis showed that maxillary and mandibular inter-canine widths increased by 2.97 ± 1.31 mm and 2.39 ± 1.32 mm, respectively, while maxillary and mandibular second primary molar buccal cusp widths increased by 1.67 ± 0.85 mm and 1.26 ± 1.11 mm, respectively (*P* < 0.001). This finding aligns with the mechanism of action of EA—the sustained force generated by the recovery of the silicone after elastic deformation acts directly on the clinical crowns, driving buccal tooth movement. Ramirez-Yañez et al. [20] treated Class II malocclusion children (aged 6–9 years, treatment duration 10 months) with a prefabricated T4K EA, observing increases in maxillary inter-canine width of 2.1 mm and inter-molar width of 1.3 mm. These magnitudes are largely comparable to those in the present study, suggesting common patterns of dental expansion with EA treatment during mixed dentition. However, that study was based on plaster model measurements, which cannot distinguish dental from skeletal expansion nor assess adaptive alveolar bone remodeling. In contrast, CEA, as a customized appliance, better conforms to the patient’s arch form, providing more precise points of force application and more stable, long-lasting stress distribution [12, 17, 19].

This study found that CEA induced notable maxillary dentoalveolar change. Maxillary alveolar crest width increased by 1.39 ± 1.09 mm, which was largely comparable to the corresponding cusp-level expansion (1.67 ± 0.85 mm), suggesting effective transmission of expansion to the maxillary alveolar bone. In contrast, the mandibular alveolar crest width showed a minimal increase of 0.38 ± 0.75 mm, which was substantially smaller than the corresponding cusp-level expansion (1.26 ± 1.11 mm) and had a 95% CI lower bound close to zero (0.10 mm). Given the minimal magnitude of this change, the lower bound of its 95% CI close to zero, and the lack of consistent statistical significance across both centers (*P* = 0.129 in Hangzhou center), the clinical significance of mandibular alveolar bone change remains uncertain. These findings should be regarded as preliminary and hypothesis-generating rather than confirmatory. Nevertheless, the presence of measurable dentoalveolar change in both arches—albeit more pronounced in the maxilla—suggests that CEA can induce adaptive dentoalveolar change beyond purely dental tipping. Mechanistically, this effect may be conferred by CEA’s unique design. The appliance encapsulates the clinical crowns and extends to the linguo-palatal alveolar bone, meaning that the sustained force generated by the silicone after elastic recovery acts not only on the clinical crowns but also directly on the alveolar bone. Concurrently, the buccal shields physically isolate abnormal buccinator and masseter muscle pressure, while the tongue ramp is thought to guide the tongue to its correct position. Collectively these features may contribute to a rebalancing of orofacial muscle forces, allowing effective transmission of mechanical stimuli to the alveolar bone to produce expansion. However, these mechanistic interpretations are speculative and require further experimental validation. This mode of action differs fundamentally from that of clear aligners, which primarily achieve expansion through force application to the clinical crowns via membrane deformation, leading to tooth tipping or bodily movement [21–24]. Notably, a recent CBCT study of mixed dentition children [8] found that after a mean treatment duration of 18.3 months with clear aligners, posterior alveolar bone width increased by 2.51 ± 1.71 mm, suggesting that clear aligners may also induce a degree of alveolar bone remodeling. Nevertheless, due to the longer treatment duration and the lack of a blank control group, the observed skeletal effects in that study cannot be completely separated from the contribution of natural growth. On the other hand, unlike rapid palatal expanders (RPE), which achieve skeletal expansion by forcefully opening the midpalatal suture [25], CEA may act through a different mechanism, possibly involving a hypothesized physiologic mode of expansion rather than forced basal bone widening. A CBCT study by Zhang et al. [25] on RPE showed that although the device achieves significant basal bone widening, it is accompanied by the risk of buccal alveolar bone resorption. The synergistic “dental movement + dentoalveolar remodeling " may allow CEA to avoid the potential risk of bone resorption while offering a more physiologically sound early treatment option for managing transverse deficiency in mixed dentition children. However, this comparative interpretation is based on theoretical considerations and was not directly tested in this study.

The absence of significant changes in maxillary or mandibular basal bone width warrants further discussion. This result may be attributed to several factors. First, the mechanical mechanism of CEA may differ from that of RPE. While RPE generates heavy, intermittent forces to open the midpalatal suture [25, 26], CEA delivers light, continuous forces that are closer to physiological levels, designed to guide dentoalveolar remodeling rather than to force basal bone expansion. Second, the anatomical characteristics of the jaws play a role: the midpalatal suture, though not completely fused in mixed dentition, requires stronger and more direct forces to open than those delivered by CEA; the mandibular symphysis undergoes bony fusion in early infancy and lacks the expansion potential of the midpalatal suture. This also partly explains why mandibular basal bone width did not increase following CEA treatment, consistent with the observation that mandibular changes with functional appliances like Twin-block primarily involve condylar growth and sagittal ramal remodeling rather than transverse basal bone widening [27]. Third, regarding the treatment duration, the mean treatment duration in this study was 8.2 months. Whether a longer treatment duration would eventually induce measurable skeletal changes remains an open question that requires further investigation. Taken together, these findings suggest that CEA may primarily promote dental and dentoalveolar changes rather than skeletal expansion. The indications for CEA should focus on cases where dental expansion and adaptive dentoalveolar change are sufficient to correct the transverse deficiency; for cases of skeletal transverse deficiency requiring significant basal bone widening, bone-borne expanders such as RPE remain the preferred choice. However, this interpretation is based on a theoretical comparison of appliance designs, rather than direct evidence from a comparative study, and should therefore be regarded as speculative.

Another methodological feature of this study is its two-center design. Compared with single-center studies, the two-center design offers several advantages: First, including patients from different clinical institutions expands the sample source, reduces the limitation on result generalizability caused by selection bias in a single center, and enhances the applicability of the conclusions. Second, both centers used identical treatment protocols, inclusion criteria, and measurement protocols. Subgroup analysis showed highly consistent results across centers, confirming the reproducibility of CEA’s expansion effects in different clinical environments—an important finding for the clinical promotion of this novel appliance. Third, the consistency of the results reinforces the robustness of the study conclusions, reducing the risk of false-positive or false-negative findings due to operational differences at a single center. However, it should be noted that CBCT equipment models differed between the two centers—both from the same manufacturer (KaVo, OP 3D Pro vs. X-trend)—and subtle inter-device differences cannot be completely excluded. To minimize this potential bias, all scans were performed with standardized low-dose parameters (90 kV, 5 mA, FOV 12 × 15 cm, MAR activated), and all CBCT data were reconstructed and measured by a single investigator using the same software and measurement protocols.

Second primary molars are generally considered to be less stable reference structures during the mixed dentition period, as they undergo physiological root resorption and eventual exfoliation. Nevertheless, under the specific conditions of the present cohort, they remain a reasonable and reliable reference choice for several reasons. First, regarding crown stability, the mean age of the patients at baseline was 8.3 ± 1.2 years, and the mean treatment duration was 8.2 ± 1.3 months, resulting in a mean post-treatment age of approximately 9.1 years — well before the peak exfoliation period of the second primary molars (typically 10–12 years). Nearly all second primary molars remained clinically stable without mobility or exfoliation, indicating good crown stability throughout the study period. Second, regarding root apex localization, at the mean age of 8.3 years, the majority of second primary molar root apices remained sufficiently intact on T0 CBCT images to allow reliable identification, and even at T1, most remained identifiable; in cases where root resorption had progressed, the measurement was performed at the corresponding anatomical level determined from the T0 scan to ensure consistency between time points. Third, the alternative of using first permanent molars was carefully considered but was not adopted, as their eruption status varies considerably at baseline and their positions change continuously with eruption, making them less stable as reference landmarks. Based on these considerations, the second primary molars remain a reasonable and reliable reference landmark for posterior width measurements under the specific conditions of this study.

This study has several limitations. First, as a retrospective study without an untreated control group, the contribution of natural growth to the observed width increases cannot be completely excluded. However, longitudinal data from untreated mixed-dentition children [28] suggest that annual increases in arch width are minimal (0.1–0.3 mm), whereas the changes observed in this study (2.97 mm for inter-canine width and 1.67 mm for molar width) substantially exceed expected growth. In addition, no comparison was made with a gold-standard treatment such as RPE. Given that CEA is a relatively new appliance, the primary objective of this exploratory study was to characterize the nature and magnitude of CEA-induced expansion (dental vs. dentoalveolar vs. skeletal), rather than to compare its efficacy against an established alternative. Moreover, the inclusion of patients with confirmed compliance reflects a practical limitation inherent to removable appliance studies, where adequate wear time is essential for treatment response. Nevertheless, future prospective randomized controlled trials with appropriate compliance monitoring protocols and longer follow-up periods are warranted to compare CEA with other expansion appliances. Second, no formal a priori sample size calculation was performed. To assess the adequacy of the sample, we performed a post-hoc power analysis based on the primary outcome measure (MSPM-ACW), which indicated that the sample size (*n* = 31) was sufficient to detect the primary effect. However, post-hoc analysis cannot substitute for a priori calculation, and future prospective studies with formal sample size estimation are needed to confirm these findings. Third, the use of two different CBCT models (both KaVo, OP 3D Pro and X-trend) may have introduced minor inter-device variability. Although standardized scanning parameters, a single investigator, and consistent software were applied to minimize this potential bias, subtle differences between the two devices cannot be completely excluded. Fourth, although the second primary molars were selected as reference landmarks for reasons discussed above, their crown and root stability remains a legitimate concern. Future studies should carefully consider whether second primary molars or first permanent molars are more appropriate as reference landmarks in mixed dentition analyses.

## Conclusions

This exploratory study suggests that CEA may expand the maxillary and mandibular arches in mixed dentition children with maxillary transverse deficiency. The expansion effect in the maxilla primarily involves dental movement and adaptive dentoalveolar change, without significant deep basal bone changes. In the mandible, significant dental expansion was observed at the cusp level, while the alveolar bone changes were minimal and should be interpreted cautiously. The high consistency of results between the two centers supports the reproducibility of this therapeutic effect. CEA may be considered a potential option for mixed dentition patients with mild-to-moderate arch constriction, although future confirmatory studies are needed to validate these preliminary findings.

## Data Availability

The datasets generated and/or analysed during the current study are available from the corresponding author on reasonable request.
